# *Medicago truncatula* ABCG10 is a transporter of 4-coumarate and liquiritigenin in the medicarpin biosynthetic pathway

**DOI:** 10.1093/jxb/erx059

**Published:** 2017-03-28

**Authors:** Wanda Biała, Joanna Banasiak, Karolina Jarzyniak, Aleksandra Pawela, Michał Jasiński

**Affiliations:** 1 Department of Natural Products Biochemistry, Institute of Bioorganic Chemistry, Polish Academy of Sciences, Poznan, Poland; 2 Department of Biochemistry and Biotechnology, Poznan University of Life Sciences,Poznan, Poland

**Keywords:** ABC transporters, biotic stress, liquiritigenin, *Medicago truncatula*, medicarpin, phenylpropanoid pathway, 4-coumarate

## Abstract

The ABCG10 protein of the model legume *Medicago truncatula* is required for efficient *de novo* production of the phenylpropanoid-derived phytoalexin medicarpin. Silencing the expression of *MtABCG10* results, *inter alia*, in a lower accumulation of medicarpin and its precursors. In this study, we demonstrate that the impairment of medicarpin biosynthesis can be partially averted by the exogenous application of 4-coumarate, an early precursor of the core phenylpropanoid pathway, and the deoxyisoflavonoid formononetin. Experiments conducted using HPLC/MS in a heterologous system as well as *in vitro* transport assays with labelled substrate revealed that MtABCG10 is responsible for the membrane translocation of 4-coumarate and liquiritigenin, molecules representing key branching points in the phenylpropanoid pathway. The identification of transporters participating in the distribution of precursors is an important step in understanding phenylpropanoid biosynthesis.

## Teaser

A full-size Medicago ABCG protein acts as a distributor of medicarpin early precursors and a modulator of phenylpropanoid pathway activity upon biotic stress.

## Introduction

The phenylpropanoid pathway (PP) products, called phenylpropanoids, are secondary metabolites derived from the amino acid L-phenylalanine. These highly diverse bioactive compounds exhibit a wide range of functions in plants, including lignin formation (Vogt, 2010), UV protection (Nguyen *et al.*, 2013), flower and fruit pigmentation (Dixon *et al.*, 2013), symbiotic associations (Zhang *et al.*, 2009; Nadal and Paszkowski, 2013), and defence reactions (Naoumkina *et al.*, 2007). Moreover, phenylpropanoids, especially flavonoids, affect human health and therefore have great pharmacological potential (Dixon and Pasinetti, 2010). The PP is one of the most extensively investigated metabolic pathways. The enzymatic steps involved in the biosynthesis of the major classes of phenylpropanoid compounds are well recognized (Naoumkina *et al.*, 2010). Nevertheless, the regulatory aspects associated with the redirection of metabolic fluxes and the transport and distribution of PP products are still not fully understood.

The three initial steps of the PP constitute its ‘core’ and include the conversion of L-phenylalanine to *trans*-cinnamic acid by phenylalanine ammonia-lyase (PAL), its hydroxylation to 4-coumarate catalysed by cinnamate-4-hydroxylase, and the action of the enzyme 4-coumarate:CoA ligase, which results in the formation of 4‐coumaroyl CoA. This general PP provides common precursors for all subsequent branches, leading to the biosynthesis of monolignols, stilbenes, coumarins, and (iso)flavonoids (Dixon *et al.*, 2002; Gholami *et al.*, 2014).

Legumes are especially abundant in flavonoids, and among the flavonoids, 5-deoxy(iso)flavonoids are almost exclusively present in legume species. Isoliquiritigenin is the first intermediate in this branch and arises through the cooperative activity of chalcone synthase and chalcone reductase. It can be further metabolized by chalcone isomerase to form liquiritigenin (Dixon *et al.*, 2002). The latter is a precursor for the biosynthesis of 5-deoxyflavonoids produced by flavone synthase II and 5-deoxyisoflavonoids produced by isoflavone synthase (IFS) (Dixon *et al.*, 2002).

Enzymes engaged in the PP are encoded by genes with multiple copies in plant genomes. The strict temporal and spatial expression of genes encoding particular isoforms, in addition to their differentiated substrate affinity and ability to form enzymatic complexes, determines the direction of carbon flow from common precursors towards the target products (Dixon *et al.*, 2002). Unravelling the role of phenylpropanoid transport in the regulation of the PP is a fundamental step in understanding such complex biosynthetic processes.

*In planta* phenylpropanoids, such as flavonoids, can be translocated between cell compartments as well as within and between plant tissues. Moreover, most flavonoid aglycones can be released into the rhizosphere, where they contribute to interactions with other plants and microorganisms (Hassan and Mathesius, 2012). Based on current knowledge, three mechanisms for flavonoid translocation have been proposed: (i) vesicle trafficking, (ii) glutathione S-transferase-aided, and (iii) membrane protein-mediated transport. The latter mechanism is directed by proteins belonging to the multidrug and toxic compound extrusion (MATE) and ATP-binding cassette (ABC) families (Zhao, 2015).

The ABC proteins constitute one of the largest families of primary active transporters that utilize ATP hydrolysis as a source of energy to transfer molecules through biological membranes. Most of them share common structural features, such as the presence of two characteristic modules, a membrane-spanning domain (TMD), and a cytosolic region containing an ABC transporter-specific nucleotide-binding domain (NBD). The ABCs can be distinguished as half-size and full-size proteins. The half-size proteins contain only the NBD and TMD (NBD-TMD), whereas full-size transporters contain two NBDs and two TMDs [(NBD-TMD)_2_] encoded by one gene (Kang *et al.*, 2011). Additionally, based on domain organization and phylogenetic relationships, the ABCs have been grouped into eight major subfamilies (A–H) (Verrier *et al.*, 2008).

While the ABC transporters are broadly distributed in all living organisms, they are especially abundant in the plant kingdom, which is rich in secondary metabolites. These secondary metabolites, derived from various pathways, are potential substrates for plant ABC proteins (Jasiński *et al.*, 2001; Verrier *et al.*, 2008; Jasiński *et al.*, 2009; Lu *et al.*, 2015; Marczak *et al.*, 2010). It has been proposed that members of the multidrug resistance-associated protein (ABCC/MRP) and pleiotropic drug resistance (ABCG/PDR) subfamilies can be involved in intra- and intercellular flavonoid translocation, respectively (Zhao and Dixon, 2009). In *Zea mays*, vacuolar anthocyanin accumulation is dependent on the tonoplast-localized protein ZmMRP3 (Goodman *et al.*, 2004). Similarly, ABCC1 from *Vitis vinifera* is involved in the co-transport of anthocyanidin 3-O-glucosides (malvidin 3-O-glucoside) and free glutathione into the vacuole (Francisco *et al.*, 2013). Biochemical studies using plasma membrane vesicles from soybean (*Glycine max*) have provided evidence that the transport of the isoflavonoid genistein displays characteristics typical for an ABC protein and suggest that this process is conducted by a G subfamily member (Sugiyama *et al.*, 2007, 2008). Among the molecules translocated by ABCG transporters, there are also simple phenylpropanoids, containing only the C_6_C_3_ phenylpropane skeleton. For instance, *p*-coumaryl alcohol is transported by ABCG29 in *Arabidopsis thaliana*, and dysfunction of AtABCG29 leads to a decrease in lignin subunits (Alejandro *et al.*, 2012). Another group of ABCG phenylpropanoid substrates are coumarins. Recently, it was shown that, upon iron deficiency, scopoletin transport to the external medium requires ABCG37 activity (Fourcroy *et al.*, 2014).

We have previously shown that the full-size ABCG transporter MtABCG10 from *Medicago truncatula* modulates the *de novo* biosynthesis of the pterocarpan phytoalexin medicarpin, derived from the 5-deoxyisoflavonoid branch. The silencing of *MtABCG10* expression resulted, *inter alia*, in a lower amount of medicarpin and its intermediates in roots as well as in root exudates, suggesting a disturbance in the early stages of medicarpin biosynthesis (Banasiak *et al.*, 2013). Here, we demonstrate that MtABCG10 is a transporter of medicarpin precursors, namely 4-coumarate and liquiritigenin. We introduce MtABCG10 as a modulator of carbon flow in the PP.

## Materials and methods

### Plant material

*Medicago truncatula* (Jemalong J5) seedlings were germinated on water-saturated Whatman paper in Petri plates and grown under controlled greenhouse conditions with a mean temperature of 22°C, 50% humidity, and a 16 h photoperiod.

Hairy root cultures were initiated from *M. truncatula* roots after the infection of a radicle with *Agrobacterium rhizogenes* strain Arqua1 (http://www.noble.org/medicagohandbook). The hairy root cultures were grown as previously described (Banasiak *et al.*, 2013). The explants of the hairy roots were transferred onto fresh medium every 3 weeks.

*Nicotiana tabacum* Bright Yellow 2 (BY2) suspension cell cultures (Nagata *et al.*, 1992) were grown in Murashige and Skoog medium supplemented with KH_2_PO_4_ (370 mg L^−1^), myoinositol (100 mg L^−1^), thiamine (1 mg L^−1^), 2,4-dichlorophenoxyacetic acid (0.2 mg L^−1^), and saccharose (30 g L^−1^) in the dark at 26°C on an orbital shaker (130 rpm) and were diluted 1:5 every week.

### Fungal elicitor and phenolic compound treatment

A cell wall oligosaccharide elicitor from *Phoma medicaginis* was prepared as previously described (Hahn *et al.*, 1992). The elicitor concentration was determined by the phenol/sulfuric acid method (Fry, 1994). The concentration used in experiments was 25 µg mL^−1^. Seven-day-old Medicago seedlings were transferred to solid 0.5× Gamborg’s medium supplemented with 4-coumarate (300 µM), isoliquiritigenin (100 µM), liquiritigenin (100 µM), formononetin (100 µM), or the fungal elicitor (25 µg mL^−1^). DMSO or water was used as a control. Samples were collected at the following time points: 0.5, 2, and 4 h for phenolic compound treatments; and 1, 2, 4, and 48 h for the elicitor treatment. The collected material was used for the quantitative RT-PCR analyses.

### Quantitative RT-PCR analyses

RNA was isolated from 7-day-old Medicago seedling roots with an RNeasy Extraction kit (Qiagen). Then, cDNA was generated from total RNA (500 ng) by reverse transcription using Omniscript (Qiagen), according to the provided protocol. For material treated with phenolic compounds, droplet digital PCR was performed with the QX200 Droplet Digital PCR (ddPCR™) System (Bio-Rad) using EvaGreen. For material treated with the fungal elicitor, real-time analyses were performed in a CFX Connect Real-Time System machine (Bio-Rad) using SYBR Green. *Actin* was used as a reference gene for normalization. For the real-time PCR assays, the gene expression levels were determined by the ΔΔCt method. For primer sequences, see Supplementary Table S1, available at *JXB* online.

### Genetic constructs and plant transformation

Genomic DNA fragments corresponding to the promoter regions of *MtABCG10*, *PALs 1–6*, and *ISFs 1–3* were amplified by PCR (for primers, see Supplementary Table S1). The PCR products were cloned into the following binary vectors: (i) pPR97, carrying the *β-glucuronidase (gusA*) reporter gene (Szabados, 1995), by restriction/ligation using restriction sites for *Bam*HI, *Eco*RI, and/or *Hin*dIII; and (ii) pPLV04_v2, carrying a *GFP* reporter gene tagged with a nuclear localization signal (NLS), by ligation-independent cloning (De Rybel *et al.*, 2011). The binary vectors pPR97 and pPLV04_v2 were transferred into *A. rhizogenes* strain Arqua1. The *M. truncatula* transformation was performed according to the protocol described in the Medicago handbook (https://www.noble.org/medicago-handbook/). Transgenic hairy roots carrying GUS reporter constructs were stained using 5-bromo-4-chloro-3-indolyl-β-D-glucuronide, as previously described (Gallagher, 1992).

The cDNA fragment (3540 bp) and the genomic DNA fragment (1575 bp) of *MtABCG10* were amplified by PCR (for primers, see Supplementary Table S1). Overlap extension PCR was conducted using primers containing sites recognized by the restriction enzymes *Asc*I and *Pac*I, and a mixture of the *MtABCG10* cDNA and genomic DNA fragments was used as the template. The PCR product was cloned by restriction/ligation into the binary vectors pMDC43 and pMDC32 (Curtis and Grossniklaus, 2003). The pMDC43 vector containing the *p35S::GFP-MtABCG10* construct was introduced into a BY2 suspension cell culture using a Bio-Rad Biolistic PDS 1000/He gene gun. Forty-eight hours after the transformation, BY2 cells with transient expression of the fusion protein GFP::MtABCG10 were observed by laser scanning confocal microscopy (Leica TCS SP5). The plasma membrane and nuclei were stained with FM4-64 and DAPI, respectively. *A. tumefaciens* strain AGL1 (Hellens *et al.*, 2000) carrying the *p35S::MtABCG10* construct in pMDC32 was used to transform BY2 cells. *A. tumefaciens* cells were added to a 5-day-old BY2 suspension cell culture and incubated for 2 days in the dark with agitation (130 rpm) at 22°C. The cells were collected, washed with fresh medium, and transferred onto plates with solid selective medium (hygromycin 75 mg L^−1^). Four-week-old callus cultures were used to initiate a suspension cell culture.

### Protein isolation and western blot assays

Microsomal fractions were isolated as previously described (Jasiński *et al.*, 2001) from 150 mg of Medicago hairy roots or 300 mg of BY2 cells. The proteins were separated by SDS-PAGE and transferred to a polyvinylidene fluoride membrane (Millipore) by electroblotting (semi-dry; apparatus; Bio-Rad). The membrane was incubated with a primary polyclonal antibody specific for MtABCG10 (Banasiak *et al.*, 2013) or with a primary antibody against H+-ATPase (W1G) (Morsomme *et al.*, 1998). The secondary antibody was an alkaline phosphatase-conjugated goat anti-rabbit IgG (Sigma).

### Biosynthetic pathway restoration

For the biosynthetic pathway restoration experiment, 3-week-old cultures of Medicago hairy roots were used. *MtABCG10*-silenced and empty vector control lines were transferred into liquid, modified Fahraeus medium, acclimatized for 24 h, and elicited as described by Banasiak *et al.* (2013). Subsequently, the hairy roots were washed with fresh, cold medium and incubated for 30 min with the addition of 4-coumarate (1 mM), formononetin (1 mM), or DMSO (0.3%) in the dark with agitation (130 rpm) at 4°C. After incubation, the plant material was washed three times using cold fresh medium. Finally, the hairy root lines were transferred to 23°C and incubated for 3 h in the dark with agitation (130 rpm). The plant material was collected, immediately frozen in liquid nitrogen, and used for HPLC/MS analysis.

### Transport assays in suspension cell culture

Four-day-old suspension cell cultures (overexpressing *MtABCG10* or wild type) were filtrated, washed, and suspended in fresh, ice-cold medium (12 g in 100 mL). After the addition of either 4-coumarate (1 mM), isoliquiritigenin (100 µM), liquiritigenin (100 µM), formononetin (100 µM), naringenin (100 µM), or 7,4’-dihydroxyflavone (100 µM) as a substrate, the cells were incubated for 30 min at 4°C with agitation (60 rpm). After incubation, the cells were filtered, washed with fresh, ice-cold medium, and transferred to 23°C. Samples were collected at the defined time points and analysed by HPLC/MS.

### Extraction of phenolic compounds and LC/ESI/MS analysis

Frozen plant tissues were ground to a uniform powder and extracted in 80% methanol. The dried samples were dissolved in 80% methanol and analysed by liquid chromatography–electrospray ionization–tandem mass spectrometry (LC/ESI/MS) using a Waters UPLC connected to a Bruker micrOTOF-Q mass spectrometer. The analyses were performed in a gradient mobile phase consisting of 0.5% formic acid (v/v) in water (A) and 0.5% formic acid (v/v) in acetonitrile (B). The m/z range of the recorded spectra was 50–1000. The analyses were performed in the ion-positive mode for phenolics and ion-negative mode for carboxylic acids. For details, see Staszków *et al.*, 2011.

### ^3^H-4-coumarate uptake into membrane vesicles

All procedures were performed on ice unless otherwise stated. Membranes were prepared from 20 g of filtered BY2 cells (overexpressing *MtABCG10* or wild type) following the procedure previously described (Jasiński *et al.*, 2001). The crude membrane fraction was suspended in STED10 buffer (10 mM Tris-HCl, 10 mM EDTA, 1 mM DTT, 10% sucrose, pH 7.0). A total of 150 µg of the crude membrane fraction was diluted to a final concentration of 1 µg/µL. For the uptake assay, we modified a method described by Ding *et al.* (2012). Uptake was initiated by adding a mixture containing radiolabelled ^3^H-4-coumarate (2 µM-40 mCi/mmol), MgCl_2_ (1 mM), and ATP (4 mM). The competition assay mixture was additionally supplemented with the phenolic compounds isoliquiritigenin, liquiritigenin, formononetin, naringenin, or 7,4’-dihydroxyflavone at a concentration of 10 µM. At defined time points, 1, 2, and 3 min samples were vacuum filtered through 0.45 µm cellulose-nitrate filters (Millipore) and washed immediately with ice-cold buffer with 1 µM of 4-coumarate. The filters were air-dried, and the remaining radioactivity was measured by liquid scintillation counting (Perkin Elmer MicroBeta2).

## Results and discussion

### 4-Coumarate restores the medicarpin biosynthetic pathway in *MtABCG10*-silenced Medicago hairy roots

**Fig. 1.**
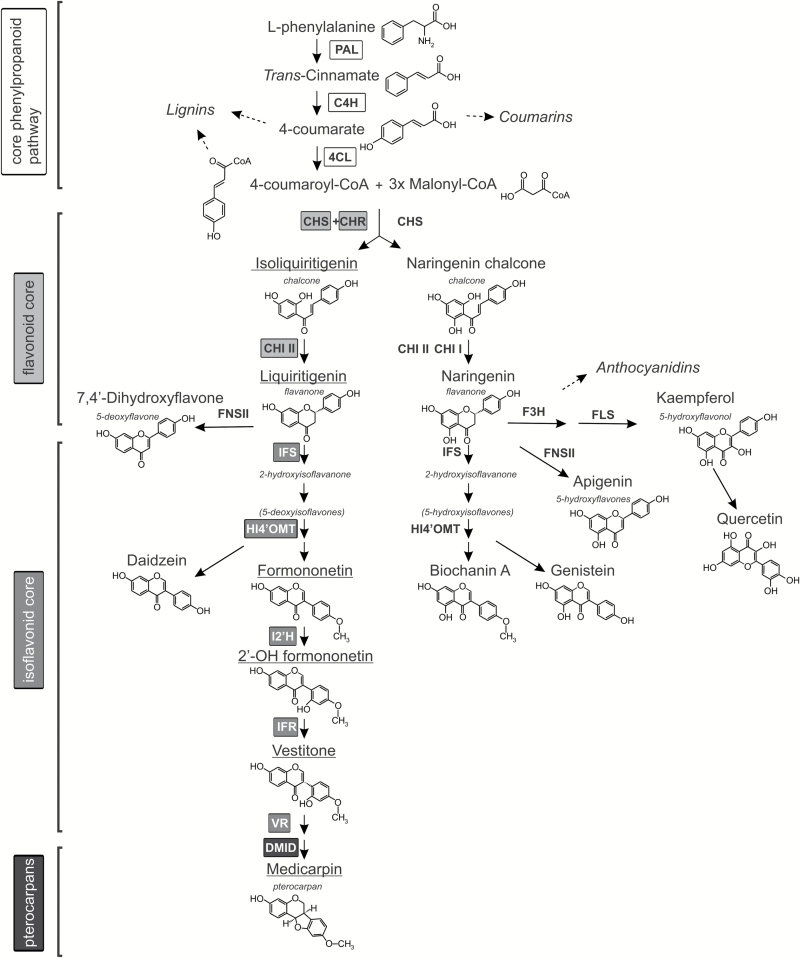
Scheme of the phenylpropanoid biosynthetic pathway in *Medicago truncatula*. The underlined compounds were affected by *MtABCG10* silencing. PAL, phenylalanine ammonia-lyase; C4H, cinnamic acid 4-hydroxylase; 4CL, 4-coumarate:CoA ligase; CHS, chalcone synthase; CHR, chalcone reductase; CHI, chalcone isomerase; IFS, isoflavone synthase; HI4’OMT, isoflavone 4’-O-methyltransferase; I2’H, isoflavone 2’-hydroxylase; IFR, isoflavone reductase; VR, vestitone reductase; DMID, 4’-methoxyisoflavanol dehydratase; F3H, flavanone 3 hydroxylase; FNSII, flavanone synthase II; FLS, flavonol synthase. The stages of the PP are presented as biosynthetic blocks, visible on the left side of the scheme.

**Fig. 2.**
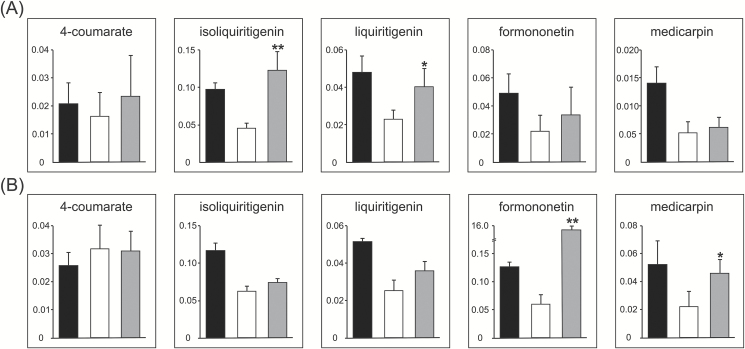
Restoration of the medicarpin biosynthetic pathway in *MtABCG10*-silenced hairy root lines. Relative levels of the selected PP products in control (black bars), *MtABCG10*-silenced (white bars), and *MtABCG10*-silenced hairy roots supplemented (grey bars) with (**A**) 4-coumarate or (**B**) formononetin. The relative amounts of the metabolites are presented as the ratio of the single-ion chromatogram peak area of the metabolite and the internal standard. The data represent the mean ± SD of five independent lines. Significant differences between supplemented and non-supplemented *MtABCG10*-silenced lines determined by Student’s *t*-test are indicated: *P < 0.05; **P < 0.01.

The pterocarpan medicarpin is a product of the PP and a major phytoalexin of *M. truncatula*. We have previously shown that the *de novo* biosynthesis of medicarpin upon biotic stress depends on the full-size MtABCG10 transporter. The lack of MtABCG10 in *M. truncatula* roots results, *inter alia*, in a lower accumulation of medicarpin and its precursors. Among these precursors, the chalcone isoliquiritigenin, from the flavonoid core of the PP (Fig. 1), is the initial precursor that is quantitatively affected by *MtABCG10* silencing (Banasiak *et al.*, 2013). To overcome the limitation of isoliquiritigenin availability and restore medicarpin biosynthesis in *MtABCG10*-silenced roots, we supplemented them with an exogenous application of 4-coumarate. 4-Coumarate is one of the earliest intermediates from the core PP, and it precedes the enzymatic steps leading to the biosynthesis of isoliquiritigenin. Because the activity of the PP is stimulated by biotic stress/elicitation (Naoumkina *et al.*, 2007), the lines used for the supplementation experiment were additionally treated with a fungal elicitor prior to the application of 4-coumarate. After 3 h of incubation in the presence of 4-coumarate, metabolites were extracted from *MtABCG10*-silenced and control hairy root lines and analysed by HPLC/MS. The target profiling and identification of phenolic compounds was based on LC retention times and high-resolution mass spectra. The HPLC/MS analysis revealed that the exogenous application of 4-coumarate onto elicited *MtABCG10*-silenced hairy root lines averted the effect of *MtABCG10* silencing and partially restored medicarpin biosynthetic pathway activity up to the intermediate liquiritigenin (Fig. 2A). Liquiritigenin represents an important branch point within the PP between 5-deoxyflavonoids and 5-deoxyisoflavonoids (Fig. 1) (Dixon *et al.*, 2002). Interestingly, exogenous application of formononetin, the first deoxyisoflavonoid to follow liquiritigenin in the medicarpin pathway, onto *MtABCG10*-silenced roots restored medicarpin biosynthesis (Fig. 2B). These data suggest that MtABCG10 dysfunction affects the PP not only at the enzymatic branch point between the core PP and flavonoids but also between the flavonoid and isoflavonoid cores.

### MtABCG10 is a 4-coumarate and liquiritigenin transporter

**Fig. 3.**
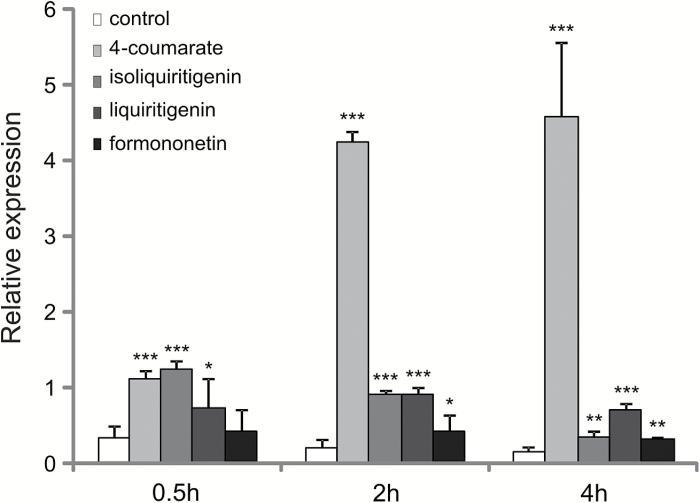
The droplet digital PCR time-course expression analysis of *MtABCG10* in *M. truncatula* seedling roots treated with selected phenylpropanoids: 4-coumarate, isoliquiritigenin, liquiritigenin, and formononetin. The transcript levels were normalized to the *Actin* gene. The data represent the mean ± SD of two independent biological experiments and two technical repeats. Significant differences from the control plants determined by Student’s *t*-test are indicated: *P < 0.05; **P < 0.01; ***P < 0.005.

**Fig. 4.**
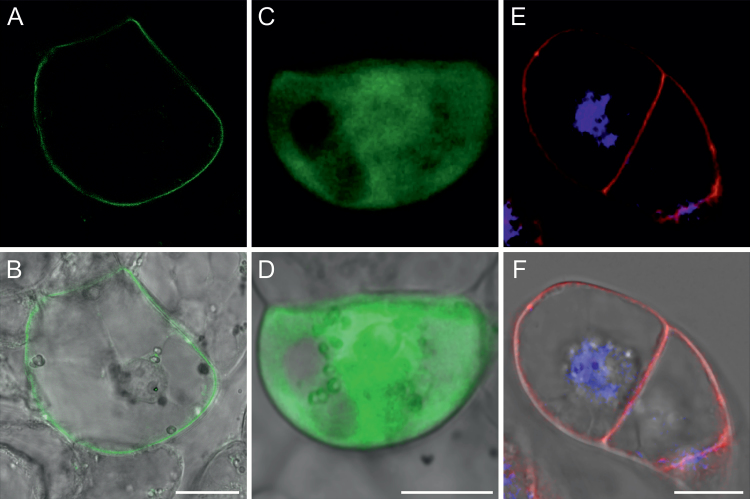
Plasma membrane localization of MtABCG10 in BY2 cells. (**A, B**) BY2 cell expressing the fusion protein GFP-MtABCG10; the GFP signal was observed at the plasma membrane. (**C, D**) Control BY2 cell expressing free cytoplasmic GFP. (**E, F**) Staining of the nucleus with DAPI and plasma membrane with FM4-64. Bars = 20 µm.

**Fig. 5.**
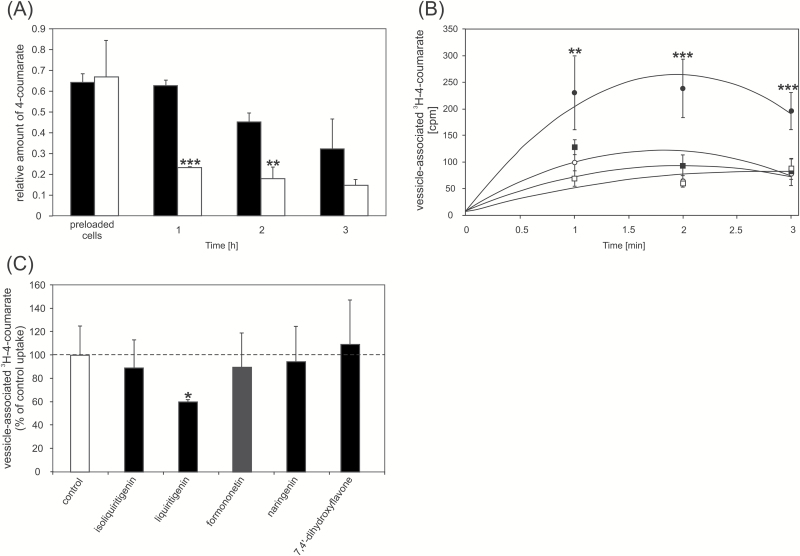
4-Coumarate transport assays in BY2 cells and cell-derived vesicles. (**A**) The 4-coumarate efflux from BY2 control (black bars) and *MtABCG10*-overexpressing (white bars) cell lines monitored by HPLC/MS. The relative amounts of the metabolite are presented as the ratio of the single-ion chromatogram peak area of the metabolite and the internal standard. The values represent the mean of three independent experiments ± SD. Significant differences between control and overexpressing lines determined by Student’s *t*-test are indicated: **P < 0.01; ***P < 0.005. (**B**) Transport of ^3^H-4-coumarate into membrane vesicles derived from BY2 cells overexpressing *MtABCG10* in the presence (closed circles) or absence (open circles) of ATP, and BY2 control cells in the presence (closed squares) or absence (open squares) of ATP. The values represent the mean of three independent experiments ± SD. The significant differences between *MtABCG10*-overexpressing lines in the presence of ATP (closed circles) in comparison to other lines/conditions determined by an ANOVA test and Tukey’s Multiple Comparison test are as follows: **P < 0.01; ***P < 0.005. (**C**) Competition between ^3^H-4-coumarate and selected phenolic compounds for transport into membrane vesicles derived from BY2 cells overexpressing *MtABCG10*. The competing substrates isoliquiritigenin, liquiritigenin, formononetin, naringenin, and 7,4’-dihydroxyflavone were used at a 10 µM concentration. The values represent the mean of six replications ± SD. The line at 100% corresponds to the vesicle-associated radioactivity 3 min after the addition of ^3^H-4-coumarate to the membrane vesicles. Significant differences between control and supplemented samples determined by Student’s *t*-test are indicated: *P < 0.05.

Among several phenolic compounds exogenously applied onto Medicago roots, 4-coumarate had the strongest effect on *MtABCG10* mRNA accumulation (Fig. 3). This observation, combined with the medicarpin biosynthetic pathway restoration data and the fact that the expression of several ABC transporters appears to be substrate-responsive (Jasiński *et al.*, 2001; Noh *et al.*, 2001; Geisler *et al.*, 2005), prompted us to determine if MtABCG10 could be a 4-coumarate transporter. To address this question, we heterologously expressed *MtABCG10* in *Nicotiana tabacum* cv. BY2 cells. The presence of MtABCG10 in BY2 cells was confirmed by western blot analysis (see Supplementary Fig. S1 at *JXB* online), and its plasma membrane localization was determined by confocal microscopy (Fig. 4). After the incubation of *MtABCG10*-expressing or control cells in the presence of 4-coumarate, the efflux of this molecule was monitored by HPLC/MS. The *MtABCG10*-overexpressing lines extruded 4-coumarate more efficiently than the control lines (Fig. 5A). To test the ATP dependence of the 4-coumarate transmembrane translocation, we conducted a transport assay using inside-out membrane vesicles isolated from BY2 cells overexpressing *MtABCG10* and ^3^H-4-coumarate. The experiment revealed that the increase of vesicle-associated radioactivity was dependent on the presence of MtABCG10 and ATP in the assay (Fig. 5B).

Using membrane vesicles and ^3^H-4-coumarate, we also performed a competition assay between 4-coumarate and other phenolic compounds from different PP branches: isoliquiritigenin, liquiritigenin, and formononetin from the medicarpin biosynthetic branch; naringenin from the 5-hydroxyflavonoid branch; and 7,4’-dihydroxyflavone, a product of the 5-deoxyflavonoid branch. Among all the tested compounds, liquiritigenin was identified as a competitor of 4-coumarate for uptake into vesicles (Fig. 5C). This observation, together with the fact that the exogenous application of 4-coumarate onto *MtABCG10*-silenced lines restored the medicarpin biosynthetic pathway only partially up to the liquiritigenin level (Fig. 2A), prompted us to determine if liquiritigenin can also be transported by MtABCG10.

**Fig. 6.**
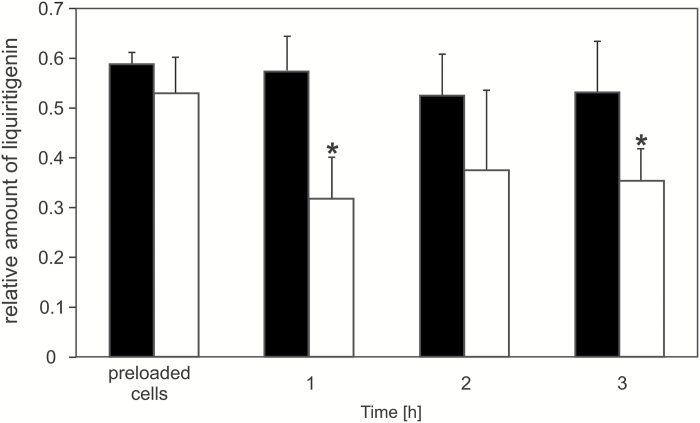
The liquiritigenin efflux from BY2 control (black bars) and *MtABCG10*-overexpressing (white bars) cell lines, monitored by HPLC/MS. The relative amounts of the liquiritigenin are presented as the ratio of the single-ion chromatogram peak area of the metabolite and the internal standard. The values represent the mean of two independent experiments ± SD. Significant differences between control and overexpressing lines determined by Student’s *t*-test are indicated: *P < 0.05.

**Fig. 7.**
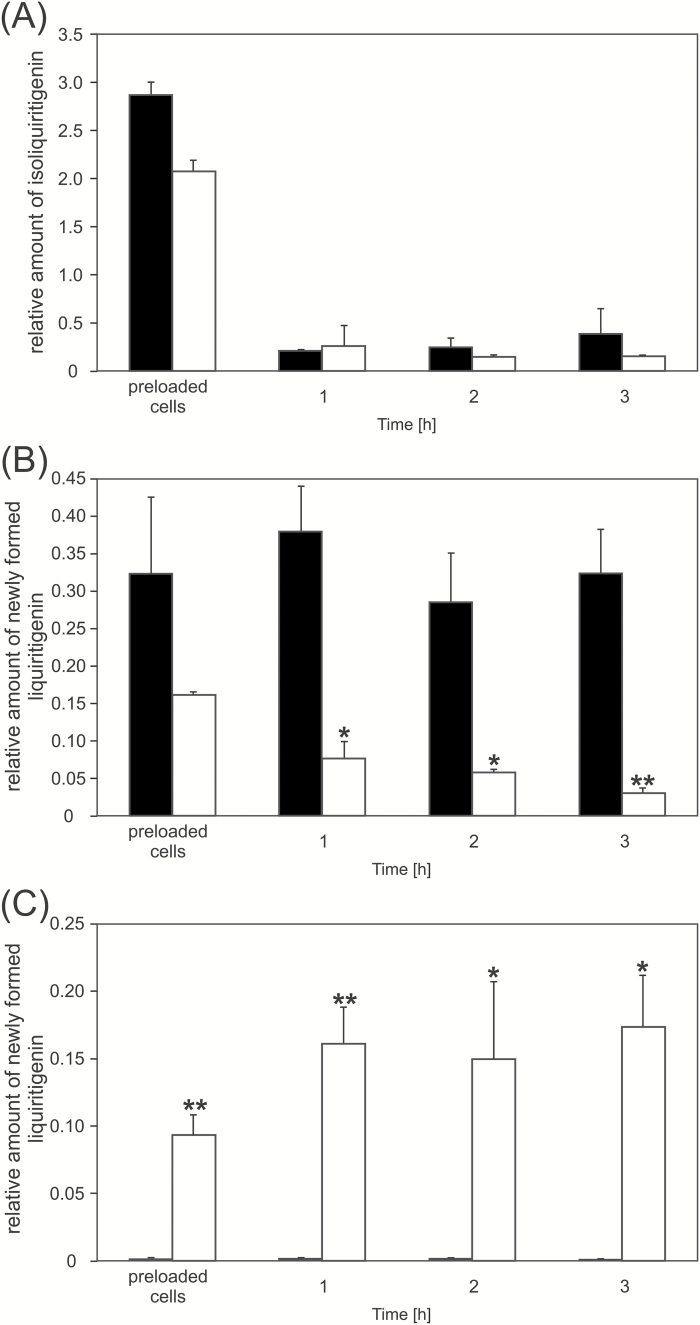
Conversion of isoliquiritigenin to liquiritigenin in control and *MtABCG10*-overexpressing cells and the MtABCG10-dependent liquiritigenin efflux. (**A**) Isoliquiritigenin amount in BY2 cells control (black bars) and *MtABCG10*-overexpressing (white bars) cell lines after the exogenous application of the isoliquiritigenin. (**B**) Amount of newly formed liquiritigenin in BY2 control (black bars) and *MtABCG10*-overexpressing (white bars) cells. (**C**) The newly formed liquiritigenin released into the medium from control (black bars) and *MtABCG10*-overexpressing (white bars) cell lines. The relative amounts of the metabolites were monitored by HPLC/MS and are presented as the ratio of the single-ion chromatogram peak area of the metabolite and the internal standard. The values represent the mean of two independent experiments ± SD. Significant differences between control and overexpressing lines determined by Student’s *t*-test are indicated: *P < 0.05; **P < 0.01; ***P < 0.005.

The liquiritigenin transport experiment conducted in BY2 cells with HPLC/MS as a detection tool revealed that liquiritigenin efflux from BY2 cells depends on the presence of MtABCG10 (Fig. 6). Further evidence that MtABCG10 acts as a liquiritigenin transporter came from an analysis of isoliquiritigenin transport. In BY2 cells, we observed the conversion of isoliquiritigenin to liquiritigenin within both control and *MtABCG10*-overexpressing cells, possibly by the nonspecific action of chalcone isomerase I or spontaneous cyclization (Fig. 7A, B) (Yu *et al.*, 2000; Shimada *et al.*, 2003; Simmler *et al.*, 2013). Interestingly, the newly formed liquiritigenin was only released to the external medium in the *MtABC10*-overexpressing lines (Fig. 7C), whereas isoliquiritigenin was not detectable in medium samples for either control or *MtABC10*-overexpressing lines (data not shown). HPLC/MS in BY2 cells showed that molecules such as formononetin, naringenin, and 7,4’-dihydroxyflavone are not transported in a MtABCG10-dependent manner (see Supplementary Fig. S2 at *JXB* online). The obtained results allow us to conclude that MtABCG10 is a transporter of precursors for medicarpin biosynthesis, namely 4-coumarate and liquiritigenin.

Finding more than one substrate for MtABCG10 was not unexpected. Plant ABCGs have been shown to possess multiple substrates, which can be illustrated by AtABCG36 (PDR8/PEN3) (Stein *et al.*, 2006; Kim *et al.*, 2007; Strader and Bartel, 2009; Lu *et al.*, 2015) or NtPDR1 (Crouzet *et al.*, 2013). Moreover, previous studies on yeast ABCGs revealed the existence of multiple substrate-binding sites in the translocation path of such transporters, which allow for the translocation of numerous, unrelated compounds (Jungwirth and Kuchler, 2006). This property of the yeast ABCG subfamily was emphasized by its former name, pleiotropic drug resistance (PDR) proteins (Verrier *et al.*, 2008). However, our observation that only certain phenolic compounds from the medicarpin biosynthesis pathway were translocated by MtABCG10 may support the assumption that plant ABCGs have higher substrate selectivity than their yeast homologues. This might be related to the sophisticated chemical system expressed by plants that provides a reliable mechanism to control various aspects of the plant (Bailly, 2014).

### MtABCG10 and the phenylpropanoid pathway

Until now, full-size plant ABCGs were thought to be engaged in the transport of the end products of secondary metabolic pathways, rather than the transport of intermediates. For example, PDR1 from *N*. *plumbaginifolia* participates in the secretion of the diterpene sclareol onto the leaf surface to protect the plant from biotic threats (Jasiński *et al.*, 2001). Similarly, its homologue from *N. tabacum* was shown to transport several terpenoids, including sclareol, manool, and cembrene (Crouzet *et al.*, 2013). AtABCG36 (PDR8/PEN3) was proposed as a transporter of indole-type active end product(s) with antimicrobial properties (Lu *et al.*, 2015). Several ABCGs have also been implicated in the transport of phenolic target compounds through biological membranes. This can be exemplified by AtABCG29, which is an exporter of the lignin monomer *p*-coumaryl alcohol (Alejandro *et al.*, 2012), as well as AtABCG37, which mediates scopoletin secretion (Fourcroy *et al.*, 2014). The presented MtABCG10 data provide evidence for the involvement of the full-size ABCG protein in the translocation of compounds from the early stages of the PP. Interestingly, both compounds transported by MtABCG10, 4-coumarate and liquiritigenin, are located at two important branch points of the PP. 4-Coumarate constitutes a branch point leading to distinct pathways for the formation of monolignols, coumarins, and (iso)flavonoids (Dixon *et al.*, 2002). Liquiritigenin is an immediate substrate for the biosynthesis of 5-deoxyflavonoids, which in Medicago are related to signalling in symbiotic interactions (Zhang *et al.*, 2009), and 5-deoxyisoflavonoids associated with defence responses (Naoumkina *et al.*, 2010).

To examine a spatial separation of PP biosynthetic stages and propose MtABCG10 as a precursor distributor during biotic stress from the general phenylpropanoid and flavonoid pathways to the isoflavonoid phytoalexin route, we decided to identify and determine the spatial expression patterns of *M. truncatulaPAL and IFS multigene family* members. These structural genes, representing two enzymatic branch points between primary and secondary metabolism and between flavo- and isoflavonoids, respectively, can be differentially regulated depending on the developmental status of the plant, the type of tissue, and environmental stimuli. Moreover, particular isoforms can be engaged in the production of distinct classes of phenylpropanoids (Dixon *et al.*, 2002). For example, *A*. *thaliana* has four PALs, some of which are involved in lignin biosynthesis, while others are more specific for flavonoids (Olsen *et al.*, 2008; Huang *et al.*, 2010). In the most recent release of the Medicago Genome Annotation Mt4.0v1, we identified six genes encoding PAL and three encoding IFS (Supplementary Table S2 at *JXB* online). Among them, we selected genes induced by elicitor treatment and potentially involved in the biotic stress response/medicarpin biosynthesis.

**Fig. 8.**
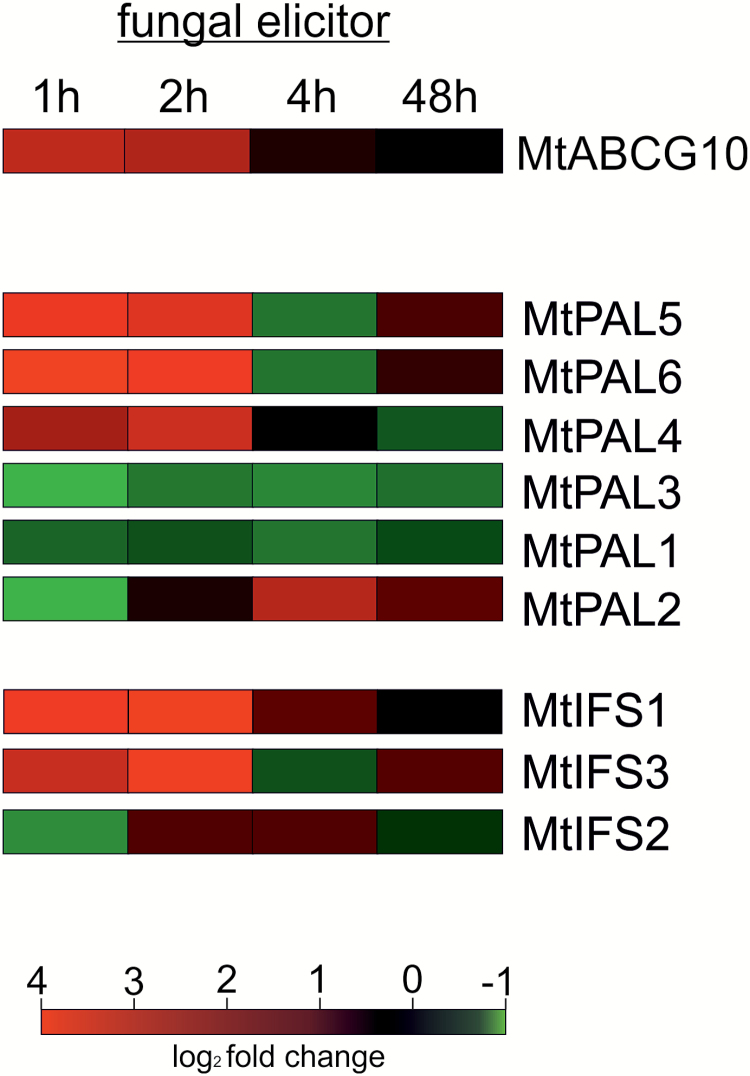
Heat map of the real-time PCR analyses of the expression of *MtABCG10*, *MtPAL*s, and *MtIFS*s in Medicago seedlings treated with a fungal elicitor. The colour indicates the fold-change value converted into a log_2_ scale, compared to the untreated control. The data represent the mean of two independent biological experiments and three technical replicates. The transcript levels were normalized to the *Actin* gene. The scale representing the relative signal intensity values is shown.

**Fig. 9.**
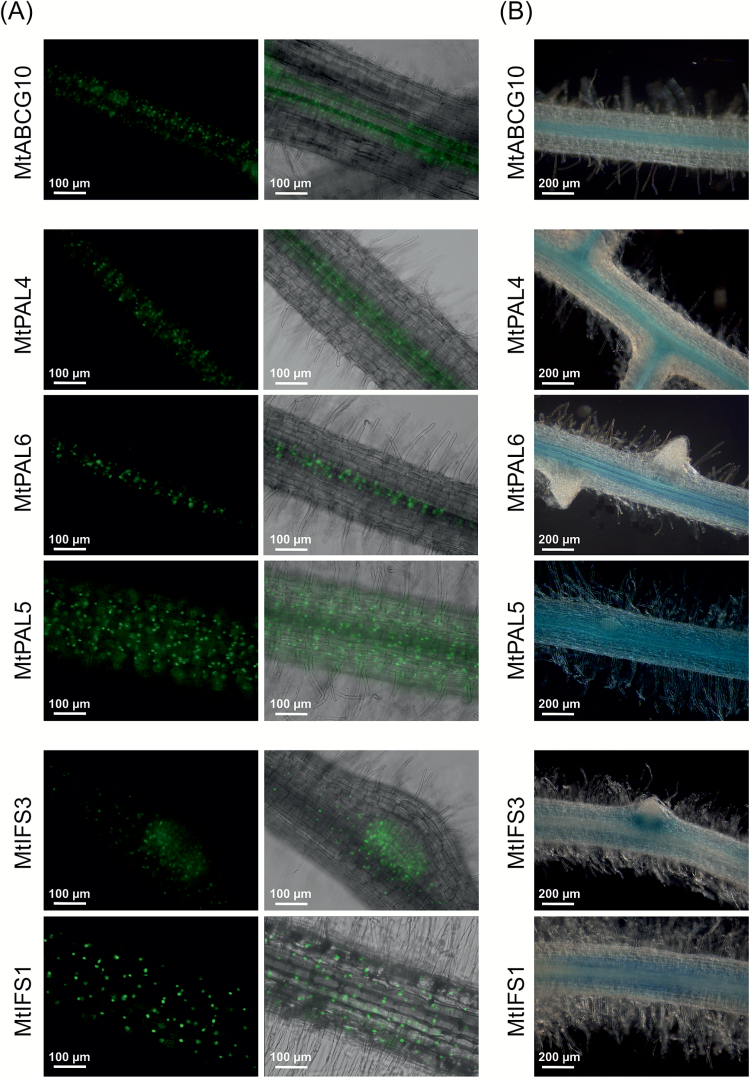
Promoter activity analyses of *MtABCG10*, *PAL*(*4,5,6*), and *IFS*(*1,3*) in transgenic *Medicago truncatula* roots. (**A**) Expression of the promoter::NLS constructs. Fluorescence images (left panel) and the merge of fluorescence and bright-field images (right panel) are shown. (**B**) Expression of the promoter::GUS reporter constructs. Transgenic roots were stained for GUS activity and visualized by light microscopy.

We found that *PAL4*, *PAL5*, and *PAL6*, as well as *IFS1* and *IFS3*, were concurrently induced with the accumulation of *MtABCG10* mRNA 1 and 2 h after elicitation (Fig. 8). Subsequently, these upregulated genes were chosen for a further examination of their promoter activity and comparison of tissue-specific expression patterns within Medicago roots. For each gene, we used two types of constructs: (i) a fusion with GFP containing a NLS (De Rybel *et al.*, 2011), and (ii) a fusion with the gene encoding gusA (Szabados, 1995). The signals were visualized by fluorescence and light microscopy, respectively (Fig. 9). The elicitor-induced genes, namely *MtABCG10* as well as individual *PAL* and *IFS* isoforms, have different spatial expression patterns. The obtained images showed that *PAL4* and *PAL6*, similar to *MtABCG10*, are expressed mainly in stele, which contains the conductive tissues, whereas *PAL5* exhibits expression within the root cortex. Two induced-by-elicitation *IFS* genes (*IFS1*, *IFS3*) are localized predominantly in the cortex. The *IFS3* promoter activity was additionally detected in the lateral root formation region. Our findings suggest that the elicitor-induced isoflavone phytoalexin pathway can be spatially separated. In this context, the transport of medicarpin early precursors into other tissues where they are finalized and/or needed for defence appears probable. Interestingly, it has been proposed that terpenoid indole alkaloid synthesis also involves the successive metabolic flow of intermediates between different types of cells (Yamamoto *et al.*, 2016).

It cannot be excluded that MtABCG10 might also function to discard excess intermediates, potentially inhibiting the PP. Phenolic intermediates can affect particular stages of the PP. For example, in Arabidopsis, *PAL* expression and its corresponding protein activity can be regulated by *trans*-cinnamic acid and its derivatives by a negative control loop (Mavandad *et al.*, 1990; Yin *et al.*, 2012; Zhang and Liu, 2015). In our previous work, it was revealed that *MtABCG10* silencing does not have an impact on *PAL* and *IFS* mRNA accumulation. Therefore, the observed metabolic phenotype is not a result of pathway activity inhibition (Banasiak *et al.*, 2013).

To conclude, in this study we have identified 4-coumarate and liquiritigenin as molecules that are translocated by ABCG10, a plasma membrane transporter from *M*. *truncatula*. The data presented constitute evidence that the full-size ABCG protein is involved in the translocation of early intermediates from the phenylpropanoid biosynthetic pathway. The regulation of metabolic fluxes and the transport and distribution of PP products are still not fully understood. Our discovery provides a new way of understanding the regulatory aspect of medicarpin biosynthesis that engages active transport.

## Supplementary data

Supplementary data are available at *JXB* online.

Table S1. List of primers used in this study.

Table S2. GenBank accession numbers for the *Medicago truncatula* sequences used in this study.

Fig. S1. Western blot analysis of crude membrane proteins obtained from BY2 wild type and *MtABCG10*-overexpressing lines.

Fig. S2. The formononetin, naringenin, and 7,4’-dihydroxy flavone transport assays in BY2 cells expressing *MtABCG10*.

## Supplementary Material

Supplementary_Tables_S1-S2

Supplementary_Figures_S1-S2
