# Supplementary material for: Medicago truncatula ABCG10 is a transporter of 4-coumarate and liquiritigenin in the medicarpin biosynthetic pathway
Source: J Exp Bot. 2017 Mar 28;68(12):3231–41. doi: 10.1093/jxb/erx059 (PMC5853973; doi:10.1093/jxb/erx059)
Supplement: Supplementary_Tables_S1-S2 [file erx059_suppl_supplementary_tables_s1-s2.pdf]

## Supplementary Tables

**Table S1.** List of primers used in this study

**A. Primers for Real-Time PCR/ddPCR analyses**

| Genes | Forward primers | Reverse primers |
|---|---|---|
| *MtPAL1* | 5'- TTGATGACCCTTGTATGGAC -3' | 5'- CTTGATTTTGTTAGGACTTGC -3' |
| *MtPAL2* | 5'- ATAGATGACAGAGAAGAAATCG -3' | 5'- GGAATTTCAGAATTTCCCTTC -3' |
| *MtPAL3* | 5'- TGGTGTCAATGGTGAGTTGC -3' | 5'- CCACCTCTTTAGGCAAAAGGG -3' |
| *MtPAL4* | 5'- CTAAGAGAACACTCACAACC -3' | 5'- ACTTCCTTTGGCAAGATAGC -3' |
| *MtPAL5* | 5'- TCCTCTTCTTGAATGCTTGG -3' | 5'- CACTAAAAATTCCGGCAAAC -3' |
| *MtPAL6* | 5'- TCCTCTTCTTGAATGCTTGG -3' | 5'- GGACACACATTAGCAATACC -3' |
| *MtIFS1* | 5'- AGTACCTGGTCAACATGGAG -3' | 5'- ACAAAGTAGGAGAGACAAGC -3' |
| *MtIFS2* | 5'- GGTGAAGAGAAAATGCACCG -3' | 5'- TGAAACATCACTCATGGACC -3' |
| *MtIFS3* | 5'- ATCCAGTGCTTTGATCTGC -3' | 5'- CAACCATTGCAACATTTTGTG -3' |
| *MtABCG10* | 5`- AACTACTGTTATGTCGACCG -3` | 5`- CAACAGCGAAAATAAAGGCA -3` |
| *Mtβ-Actin* | 5'- TTCTCTCAGTACTTTCCAGC -3' | 5'- AAGCATCACAATCACTCC -3' |

**B. EcoRI (atgaattc)/BamHI (taggatcc)/HindIII (actaagctt) tagged primers for promoters activity analyses - GUS (gene specific sequences underlined)**

| Genes | Forward primers | Reverse primers |
|---|---|---|
| *MtPAL4* | 5'- atgaattcCTAAATAAACTTCAATTATACGCACC -3' | 5'- taggatccAATTACGAATGCACGAATATTTTACC -3' |
| *MtPAL5* | 5'- atgaattcACATTGGTATATCTTGTTTAGTC -3' | 5'- taggatccATCTTAATTGATAATAATTAATGTTCC -3' |
| *MtPAL6* | 5'- atgaattcGG ATG TTG GTA ACA TGA G -3' | 5'- taggatccGATCTTATATTGATACTAATAG -3' |
| *MtIFS1* | 5'- atgaattcAATGTGTAGTATTCTCATGG -3' | 5'- actaagcttGGTGTATAGCTTCTTTGTAG -3' |
| *MtIFS3* | 5'- atgaattcGAGAAATGATATTTGTACATC -3' | 5'- taggatccGGTGTATTGTAGTATATTTAC -3' |
| *MtABCG10* | 5'- atgaattcGTGTCCTTGATTAACTCACC -3' | 5'- taggatccTTTTTGTGCTGTTGTGAAAC -3' |

**C. LIC_F adapter (tagttggaatgggttcgaa)/ LIC_R adapter (ttatggagttgggttcgaa) tagged primers for promoters activity analyses - NLS:GFP (gene specific sequences underlined)**

| Genes | Forward primers | Reverse primers |
|---|---|---|
| *MtPAL4* | 5'- tagttggaatgggttcgaaCTAAATAAACTTCAATTATACGCACC -3' | 5'- ttatggagttgggttcgaaAATTACGAATGCACGAATATTTTACC -3' |
| *MtPAL5* | 5'- tagttggaatgggttcgaaACATTGGTATATCTTGTTTAGTC -3' | 5'- ttatggagttgggttcgaaATCTTAATTGATAATAATTAATGTTCC -3' |
| *MtPAL6* | 5'- tagttggaatgggttcgaaGG ATG TTG GTA ACA TGA G -3' | 5'- ttatggagttgggttcgaaGATCTTATATTGATACTAATAG -3' |
| *MtIFS1* | 5'- tagttggaatgggttcgaaAATGTGTAGTATTCTCATGG -3' | 5'- ttatggagttgggttcgaaGGTGTATAGCTTCTTTGTAG -3' |
| *MtIFS3* | 5'- tagttggaatgggttcgaaGAGAAATGATATTTGTACATC -3' | 5'- ttatggagttgggttcgaaGGTGTATTGTAGTATATTTAC -3' |
| *MtABCG10* | 5'- tagttggaatgggttcgaaGTGTCCTTGATTAACTCACC -3' | 5'- ttatggagttgggttcgaaTTTTTGTGCTGTTGTGAAAC -3' |

**D. Primers used for clonning of MtABCG10 genomic/cDNA hybrid; AscI (ggcgcgcc)/ PacI (ctacttaattaa) tagged primers (gene specific sequences underlined)**

| DNA fragment | Forward primers | Reverse primers |
|---|---|---|
| genomic part | 5'- ATGGAGGGAACAGATATATACAG -3' | 5'- GGGTCAGGCTTGATATTTGC -3' |
| cDNA part | 5'- ATGCTATCCGAGCTTTCTAG -3' | 5'- CTATCTCTTTTGGAAGTTAAAGG -3' |
| hybrid | 5'- ggcgcgccAATGGAGGGAACAGATATATACAG -3' | 5'- ctacttaattaaCTATCTCTTTTGGAAGTTAAAGG -3' |

**Table S2.** GenBank accession numbers for the *Medicago truncatula* sequences used in this study

**PAL ( L-phenylalanine ammonia - lyase)**

| Gene name | Loci | mRNA | Protein |
|---|---|---|---|
| *MtPAL1* | MTR_2g094960 | XM_013609864 | XP_013465318 |
| *MtPAL2* | MTR_5g098720 | XM_003618028 | XP_003618076 |
| *MtPAL3* | MTR_1g094780 | XM_003591829 | XP_003591877 |
| *MtPAL4* | MTR_1g064090 | XM_003590423 | XP_003590471 |
| *MtPAL5* | MTR_7g101395 | XM_013594536 | XP_013449990 |
| *MtPAL6* | MTR_7g101425 | XM_013594537 | XP_013449991 |

**IFS (isoflavone synthase)**

| Gene name | Loci | mRNA | Protein |
|---|---|---|---|
| *MtIFS1* | MTR_4g088195 | XM_013601477 | XP_013456931 |
| *MtIFS2* | MTR_4g088170 | XM_013601474 | XP_013456928 |
| *MtIFS3* | MTR_4g088160 | XM_013601473 | XP_013456927 |

**Others**

| Gene name | Loci | mRNA | Protein |
|---|---|---|---|
| *MtABCG10* | MTR_2g102670 | XM_003597771 | XP_003597819 |
| *Mtβ-Actin* | MTR_3g095530 | XM_003602497 | XP_003602545 |
