# Supplementary material for: Medicago truncatula ABCG10 is a transporter of 4-coumarate and liquiritigenin in the medicarpin biosynthetic pathway
Source: J Exp Bot. 2017 Mar 28;68(12):3231–41. doi: 10.1093/jxb/erx059 (PMC5853973; doi:10.1093/jxb/erx059)
Supplement: Supplementary_Figures_S1-S2 [file erx059_suppl_supplementary_figures_s1-s2.pdf]

Supplementary Figures

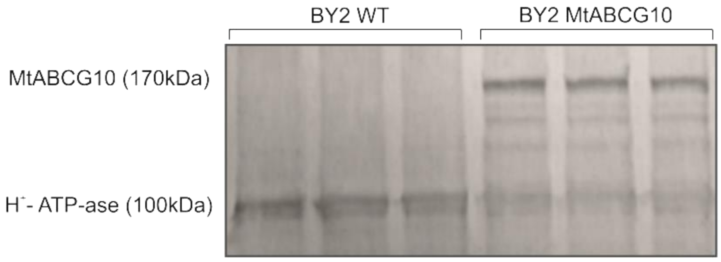

Fig. S1. Western blot analysis of the crude membranes, obtained from BY2 WT and *MtABCG10*-overexpressing lines. Antibodies used in the assay are: primary polyclonal specific for MtABCG10 (Banasiak *et al*., 2013), primary polyclonal against H+-ATPase (W1G) (Morsomme *et al.,* 1998) and the secondary alkaline phosphatase-conjugated goat anti-rabbit IgG (Sigma).

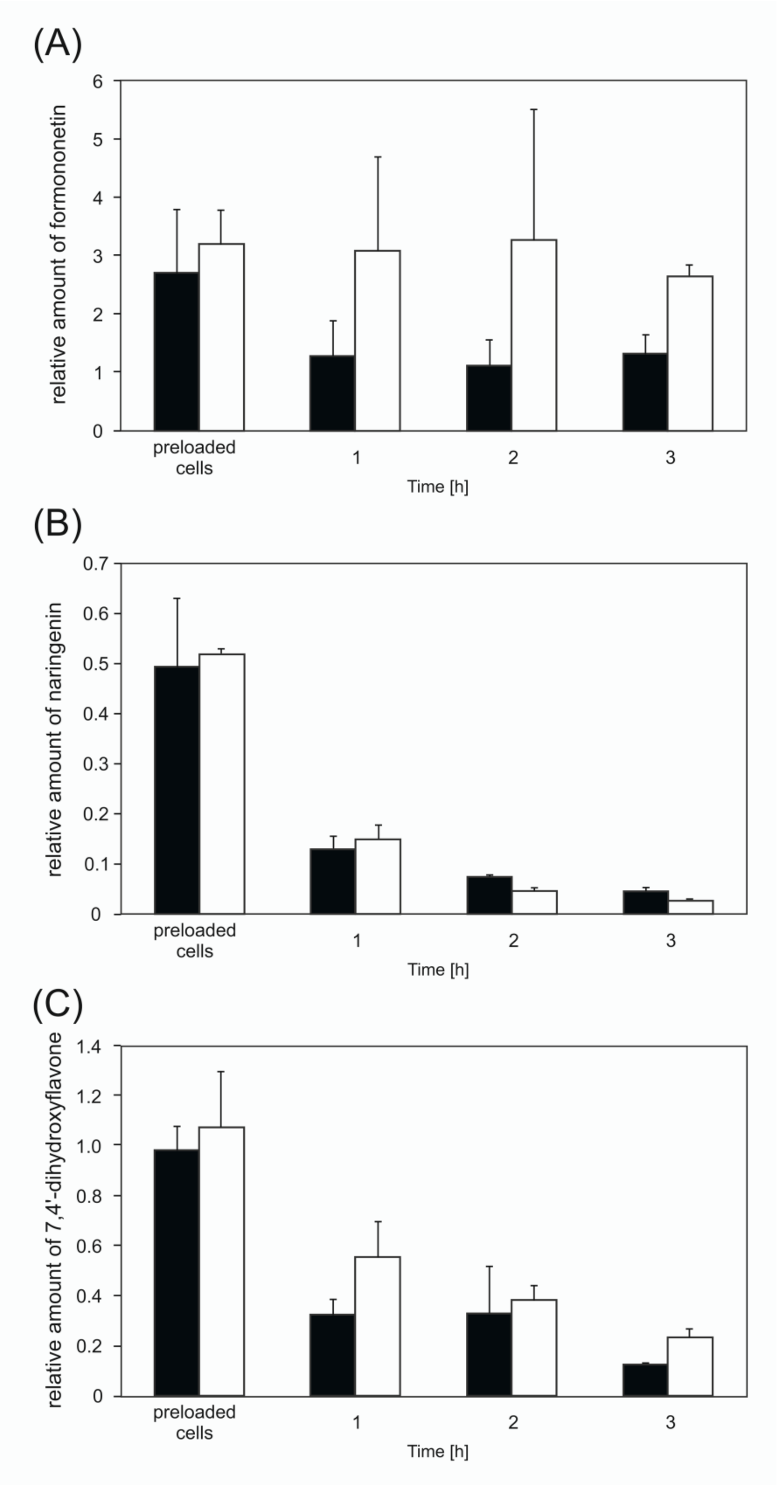

Fig. S2 Efflux of different phenolic compounds from BY2 cells. (A) Formononetin, (B) naringenin, (C) 7,4'-dihydroxyflavone efflux from BY2 control (black bars) and MtABCG10-overexpressing (white bars) cell lines monitored by HPLC/MS. The relative amounts of the metabolite are presented as the ratio of the single-ion chromatogram peak area of the metabolite and the internal standard. The values represent the mean of two independent experiments ± SD.
